# Development of breast muscle parameters, glycogen reserves, and myogenic gene expression in goslings during pre- and post-hatching periods

**DOI:** 10.3389/fphys.2022.990715

**Published:** 2022-09-13

**Authors:** De Xin Dang, Haizhu Zhou, Yujie Lou, Xiao Liu, Desheng Li

**Affiliations:** ^1^ College of Animal Science and Veterinary Medicine, Jinzhou Medical University, Jinzhou, China; ^2^ Department of Animal Resources Science, Dankook University, Cheonan, South Korea; ^3^ College of Animal Science and Technology, Jilin Agricultural University, Changchun, China; ^4^ Institute of Animal Nutrition, College of Animal Science and Technology, Northeast Agricultural University, Harbin, China

**Keywords:** gosling, breast, glycogen, myogenic gene, early-life development

## Abstract

This study aimed to better understand the development patterns of breast muscle and glycogen reserves in goslings during pre- and post-hatching periods. The timepoints for sampling were embryonic days 23 and 27 of hatching and days 1, 4, and 7 post hatching. We found that the body weight of goslings increased with age. The small intestine developed with age and remained reasonably constant on day 4 post hatching. The breast muscle development decreased with age and stayed relatively stable on day 1 post hatching. The diameter of myofiber increased prior to hatching and then decreased while hatching. The development patterns of breast muscle glycogen reserves were similar to the diameter of myofiber. In contrast, the contents of liver glycogen began to decrease before hatching and then increased rapidly after hatching. Moreover, the expression of Myf-5 increased with age. The expression of MSTN was maintained at high levels prior to hatching, dropped immediately after hatching, and then gradually increased with age. Additionally, we also observed that the glycogen content in the breast muscle was positively correlated with the diameter of the myofiber. The liver glycogen content was positively correlated to the relative weight of the breast muscle, the diameter of the myofiber, and the breast muscle glycogen content. The development pattern of the myofiber was synchronized with the change in the MSTN/Myf-5 ratio. This study provided a profile to understand the development patterns of breast muscle, glycogen reserves, and myogenic gene expression in goslings, which was beneficial to understanding the characteristics of energy reserves during the early life of goslings.

## Introduction

Geese are widely raised around the world, and they are one of the important sources of commercial meat. Muscle tissue is the most valuable edible component of geese. Goose is rich in protein and various trace elements, such as calcium, phosphorus, potassium, and sodium. However, it is low in fat and cholesterol (Xu et al., 2018). As a result, geese are a nutritious and healthy food resource.

Hatching and embryo development are the key parts of goslings’ lives. The embryonic stages before hatching are crucial for muscle development of poultry, as they determine the muscle mass in adulthood. The development of breast muscle is closely related to glycogen storage since an insufficient glycogen reserve will force the embryo to mobilize muscle proteins through the gluconeogenesis process (Uni et al., 2005). Additionally, the myogenic regulatory factor, Myf-5, the growth factor and differentiation factor-8, and MSTN play an important role in regulating the growth and development of muscles (Crist et al., 2012; Kim et al., 2020). Any improvement in the embryonic stages of goose breeding contributes to a significant increase in the efficiency and profitability of goose production. A comprehensive investigation has been conducted on the embryonic breast muscle development of chicks and ducks (Scheuermann et al., 2003; Li et al., 2010). It was reported that embryonic day 19 is the key timepoint for development of breast muscle in ducks, in which the expression levels of myogenic factor 6 and myogenin are the highest, while the expression level of MSTN is the lowest (Gu et al., 2013; Li et al., 2020). With the development of duck embryo, the breast muscle mass decreased, whereas the weight of the liver and small intestine increased on embryonic day 22 (Chen et al., 2009). Simultaneously, the glycogen content in the liver and breast muscle decreased with the increase of duck embryo age (Chen et al., 2010). Similarly, the glycogen reserves from the liver and breast muscle of broiler chicks reached their maximum concentration on embryonic day 19, while in the last 2 days of hatching, a dramatic decrease was observed (Borrebaek et al., 2007; Zhai et al., 2011a,b; Yadgary and Uni, 2012). In turkey, Moore et al. (2005) found that the cross-sectional area of the myofiber decreased as the embryo approached the hatching age. However, the embryonic development patterns in the breast muscle and glycogen reserves of goslings during pre- and post-hatching periods have not been fully understood.

Future meat production will benefit from research on the skeletal muscle development pattern of goslings. Therefore, this study was performed to investigate the breast muscle development pattern, glycogen reserves, and myogenic gene expression in goslings during pre- and post-hatching periods.

## Materials and methods

This study was approved by the Animal Care and Use Committee of Jilin Agricultural University (Changchun, Jilin, China).

### Experimental animals

A total of 150 fertilized eggs with the breed of Jilin White geese used in this study were obtained from the Geese Experimental Center of Jilin Agricultural University. All the eggs were laid on the same day and were of the same weight. Fertilized goose eggs were incubated in standard conditions in an incubator (Keyu CFZ microcomputer automatic incubator, Dezhou, Shandong). Before being moved to the incubator, eggs were pre-heated to 30°C for 12 h, disinfected with 37% formaldehyde and potassium permanganate (2:1), and distributed into incubator tray levels. The hatching period included three phases (phase 1, days 1–14; phase 2, days 15–28; and phase 3, days 29–31). During phase 1, the temperature was 38°C, and the humidity was 65%; during phase 2, the temperature was 37.5°C, and the humidity was 55%; and during phase 3, the temperature was 37.2 °C, and the humidity was 70%. Every egg was turned for 180 s every 2 h.

On embryonic day 23, eggs were examined with a candle to check the embryonated eggs in order to remove any egg that was unfertilized. A total of 120 fertilized eggs with similar weights were used for further experiments. In the present study, the sampling timepoints were embryonic days 23 and 27 of hatching and days 1, 4, and 7 post hatching. At each sampling timepoint, a total of 18 eggs were randomly selected and randomly assigned into six groups, with three repetitions per group.

After hatching, the geese were transported to the farm and distributed into cages (25 birds per cage). Birds were fed the diet (Table 1) as soon as they were placed in the cages. All birds were kept under uniform management conditions. All geese were housed in a temperature-controlled room with continuous lighting. The temperature of the room was maintained at 30°C. Birds had free access to feed and water.

**TABLE 1:** Composition and nutrient levels of the experimental basal diet (%, as-fed basis).

Ingredients, %	
Corn	60.00
Soybean meal	29.11
Wheat bran	6.00
Fish meal	2.00
Lysine-HCl	0.20
Methionine	0.23
Dicalcium phosphate	0.84
Limestone	0.82
Sodium chloride	0.30
Vitamin and trace mineral premix^a^	0.50
Total	100.00
Calculated value, %	
Metabolizable energy, MJ/kg	11.67
Available phosphorus	0.40
Analyzed composition, %	
Calcium	0.78
Crude protein	19.78
Methionine	0.50
Total sulfate amino acid	0.77
Lysine	1.08
Crude fiber	0.31
Neutral detergent fiber	1.09
Acid detergent fiber	0.35

a Provided per kg of complete diet: vitamin D_3_, 200 IU; vitamin A (retinyl acetate), 1500 mg; vitamin E (DL-α-tocopheryl acetate), 12.5 mg; vitamin K_3_, 1.5 mg; thiamine, 2.2 mg; riboflavin, 5 mg; nicotinic acid, 65 mg; folic acid, 1 mg; pantothenic acid, 15 mg; pyridoxine, 2 mg; biotin, 0.2 mg; choline, 1000 mg; Fe, 90 mg; Cu, 6 mg; Mn, 85 mg; Zn, 85 mg; I, 0.42 mg; Se, 0.3 mg; Co, 2.5 mg.

### Feed analysis

After homogeneous mixing, feed samples were collected from each dietary group. All feed samples were dried in a 70°C constant temperature oven for 72 h. Subsequently, feed samples were ground and sieved with a 1-mm sieve. According to the procedure established by the Association of Official Analytical Chemists (AOAC, 2000), the dry matter (method 930.15), crude protein (nitrogen × 6.25; method 968.06), calcium (method 984.01), and crude fiber composition (method 991.43) in the diet were analyzed. Then, the representative feed samples in each group were hydrolyzed with 6 N HCl for 24 h at 110°C. An amino acid analyzer (2690 Alliance, Waters, Inc., Milford, MA, United States) was used for determining amino acid content in the diet. In addition, the contents of neutral detergent fiber and acid detergent fiber in the diet were measured according to the method provided by Mertens (2002).

### Sample collection and measurement

Eggs were opened with surgical scissors at the blunt end of the egg to extract the embryo. Embryos or young goslings were killed by cervical dislocation. Body weight (with yolk sac) of all birds was recorded. For all time points, the intestinal contents and adherent materials were removed carefully under ice-cold saline and weighed.

The liver was removed from the carcass, and the adherent material of the liver was carefully removed under ice-cold saline. About 100 mg of liver samples was frozen as aliquots in liquid nitrogen and stored at −80°C for measurement of the liver glycogen content.

Both sides of the breast muscle were stripped and weighed after euthanasia. One side of the breast muscle sample was fixed with 10% neutral-buffered formalin for analyzing myofiber traits. Another side of the breast muscle sample was frozen as aliquots in liquid nitrogen and stored at -80 °C to measure breast muscle glycogen content and breast muscle development-related gene expression.

### Experimental parameter measurement

Breast muscle and small intestine parameters: the relative weight of breast muscle and small intestine were calculated using the following equation:

$$Organ\;index\;=\;\frac{Organ\;weight}{Live\;body\;weight}\;\times \;100\;\%.$$

### Myofiber traits analysis

After slaughtering the geese, the breast muscle samples were cut into small pieces and fixed with 10% neutral-buffered formalin for 12 h, followed by dehydration in increasing concentrations of alcohol (70%, 80%, 90%, 95%, and 100%) and xylene. Consequently, samples were embedded in paraffin and stored in an oven at 60°C. Twelve hours later, samples were removed from the oven and histological cassettes. Fragments were placed in “paper boxes” and covered with paraffin. After the paraffin solidified into blocks, the “papers” were removed, and the blocks were kept under refrigeration until the cuts were realized (Felício et al., 2013).

Serial tissue sections (3 μm thickness) were excised perpendicular to the direction of the myofibers using a cryostat. After sectioning, the paraffin section ribbon was put on the coating slide glass. Dried slides were kept in the oven at 60°C for 2 h to eliminate excess paraffin. The next step consisted of paraffin removal and slide hydration, using xylene and different concentrations of ethanol. Samples were then stained following the hematoxylin and eosin staining protocol (Felício et al., 2013).

Samples were then dehydrated again and mounted. In each specimen, the diameters of muscle fibers were measured under a light microscope equipped with a ScopePhpto (LY-WN 300, Hangzhou Scopetek Opto-Eletric Co., Ltd.).

No fewer than 150 intact, well-oriented muscle fibers’ cross sectional area of five fields of vision were measured under 40 ×objective lens. With muscle fibers assumed to be round, the muscle fiber cross-sectional area (A) was converted to diameter (D) by the formula, D = 2√A/π. The average value was calculated to represent the diameter of the muscle fibers (Liu et al., 2019).

### Glycogen reserve analysis

About 0.1 g samples of liver and breast muscle were stored at 1 ml of 8% HClO_4_, homogenized (in ice) for 45 s, and centrifuged at 7700 rpm at 4°C for 16 min for analyzing glycogen reserves by following the method provided by Foye et al. (2006). A 10-μl aliquot of the supernatant was transferred to a clean polypropylene tube, along with 0.4 ml of 8% perchloric acid and 2.6 ml of iodine color reagent made of 1.3 ml of solution A (0.26 g iodine +2.6 g potassium iodide dissolved in 10 ml of distilled water) in 100 ml of 67.8% saturated calcium chloride. All samples were read at a wavelength of 450 nm. The amount of glycogen present in the sample solution was determined by the preparation of a known glycogen standard curve.

### Muscle growth-related gene expression analysis

Total RNA was isolated from muscle samples using RNAiso reagent (TaKaRa, Dalian, Liaoning, China). The RNA integrity was assessed by electrophoresis on a 1% agarose gel containing formaldehyde. The RNA concentration was measured using a Beckman DU-640 spectrophotometer (Beckman). The sequences of primers for the genes tested were specifically designed according to the sequences located in GenBank (Table 2). The total RNA samples were purified and subjected to reverse transcription using the Takara PrimeScript RT Reagent Kit with gDNA Eraser (Takara, Dalian, China) and processed for cDNA synthesis as per the Takara PrimeScript RT instructions (Zhang et al., 2014).

**TABLE 2:** Primers used for quantitative real-time PCR.

Gene	Accession number^a^	Size (bp)^b^	Primer sequence (5′→3′)	
β-Actin	M26111	158	Forward	GCCCAGCACGATGAAGAT
			Reverse	ATT​TAC​GGT​GGA​CGA​TGG​AC
MSTN	AY448009	133	Forward	GTG​GCT​CTT​GAT​GAC​GGT​AGT
			Reverse	GCA​GTG​TGC​TGA​GGA​TTT​GA
Myf-5	KU744843	147	Forward	GCG​TTT​GAG​ACC​CTG​AAG​AG
			Reverse	TCC​CGG​CAG​GTG​ATA​GTA​GT

Abbreviation: Myf-5, myogenic factor-5; MSTN, myostatin.

a Accession number refer to Genbank (NCBI).

b PCR, product size (base pairs).

The relative expression levels of Myf-5 and MSTN genes in pectoral muscles were analyzed by RT-PCR, which was performed in a 10-μl reaction mix containing 1 μl 2 × SYBR Premix Ex Taq II (TakaRa, Dalian, China), 3 μl dH_2_O, 0.5 μl of the forward and reverse primers, and 1 μl cDNA using a Bio-Rad CFX-96 thermocycler (Bio-Rad, CA). The reaction conditions were as follows: initial denaturation at 95°C for 30 s and 44 cycles of amplification at 72°C for 30 s. The annealing was carried out for 40 s at temperatures specific to each target gene. At the end of the amplification, step-wise melting curves were performed to confirm the product specificity. The cytoskeletal protein, β-actin, was used as the internal reference. All the reactions were performed in triplicate. Sampling timepoint of the embryonic day 23 was used as the untreated control timepoint (value = 1). The formulas used for the calculations were as follows:

ΔCt(sample)=Ct(target gene)−Ct(reference gene)

ΔCt(calibrator)=Ct(target gene)−Ct(reference gene)

ΔΔCt=ΔCt(sample)−ΔCt(calibrator)

The gene expression levels of the samples were determined by the 2^−ΔΔCt^ method (Schmittgen and Livak, 2008).

### Statistical Analysis

Values from each group (three eggs) were pooled to form a sample. A total of six samples were used at each sampling timepoint for data analysis. All data were analyzed using SPSS18.0 software. The results were considered significant at *p* < 0.05. The myogenic gene expression data were analyzed using the GLM procedure. Differences among timepoints were evaluated by one-way ANOVA for multiple comparisons. The partial correlations between the development parameters of breast muscle and glycogen content were analyzed by the CORR procedure.

## Results and discussion

Geese grow fast in the early stages of life. In this study, the body weight of goslings increased with age; moreover, the small intestine gradually matured with the rapid increase in body weight (Table 3). The development of the small intestine during the late stage of incubation and the early stage after hatching is essential to optimize the growth potential of goslings (Jia et al., 2011). The small intestine has high metabolic rates, which indicates that a large amount of energy used to maintain the normal development of embryos is also used to support the development of the small intestine during the early stages of life (Rolfe and Brown, 1997). This will cause the embryo to be in a low energy state. Based on the concept proposed by Lilja (1983), during the early life of birds, there is a hierarchy for available nutrients that are allotted to “supply” tissues (muscle) and “demand” tissues (intestine). The study by Sklan (2001) indicated that energy distribution has a high priority in the intestinal growth of chicks during early life stages. Therefore, in the last few days of hatching and the first few days after hatching, the rapid growth of the small intestine induced a considerable amount of energy catabolism.

**TABLE 3:** Body weight, small intestine, and breast muscle parameters of goslings between embryonic day 23 to day 7 post hatching^a^ ^,^ ^b^.

Age^c^	Body weight, g	Relative weight of small intestine, %	Relative weight of breast muscle, %	Diameter of myofiber, mm
E23	46.85 ± 2.51^e^	7.50 ± 0.41^e^	19.18 ± 1.27^a^	6.04 ± 0.43^b^
E27	69.33 ± 2.78^d^	11.64 ± 0.47^d^	10.57 ± 0.23^b^	6.67 ± 0.16^a^
DOH	80.53 ± 10.75^c^	36.84 ± 4.69^c^	9.98 ± 0.49^b^	5.55 ± 0.22^c^
D1	80.29 ± 2.84^c^	46.53 ± 2.51^b^	6.99 ± 1.18^c^	4.66 ± 0.33^d^
D4	120.45 ± 7.21^b^	64.00 ± 4.29^a^	6.72 ± 0.58^c^	5.02 ± 0.32^d^
D7	158.96 ± 4.92^a^	62.51 ± 2.50^a^	6.15 ± 0.73^c^	5.96 ± 0.34^b^

^a-e^Different superscripts within a column indicate a significant difference (*p* < 0.05).

a The data are presented as the means ± standard deviation.

b Value presents the means of six samples (values from the three birds were pooled to form one sample) per sampling timepoint (*n* = 6).

c Age refers to embryonic day 23 (E23), day 27 (E27), day of hatch (DOH; after hatch but before feeding), and day 1 (D1), 4 (D4), and 7 (D7) post hatching.

The energy needed for embryo development is stored in the liver and breast muscle in the form of glycogen, but the quantity is limited (Givisiez et al., 2020). Glycogen as an important energy resource in maintaining the normal metabolism, and body growth during pre- and post-hatching periods of birds is crucial for development of the embryo (Moran, 2007). As reported by Zhang et al. (2016), the liver is the main energy storage organ compared with the breast muscle. Liver glycogen is the most important energy reserve to maintain embryonic development during the incubation stages, and it has been used to indicate the energy state in birds (Uni et al., 2005). In this study, the level of liver glycogen declined from embryonic day 27 to the day of hatching; however, it increased after hatching (Table 4). Correspondingly, the glycogen content in the breast muscle decreased starting from embryonic day 27 until post hatching (Table 4). This indicated that the catabolism event happens in the late term of incubation, manifested in the reduction of glycogen content during the late-term development of the embryo. Similarly, many studies reported that the concentration of liver glycogen in chicks dropped sharply during the last 2 days of hatching (Borrebaek et al., 2007; Yadgary et al., 2010; Zhai et al., 2011a; Yadgary and Uni, 2012). Chen et al. (2010) also noted that substantial consumption of body energy reserves occurred in late-term duck embryonic development. They observed a reduction of liver and breast muscle glycogen content with the age of embryos. There has not been any research which investigated the glycogen storage of geese around hatching. The results observed in this study demonstrated that goslings consumed a lot of energy in late-term embryonic development, which was probably used for supporting the development of the small intestine.

**TABLE 4:** Liver and breast muscle glycogen contents of goslings between embryonic day 23 to day 7 post hatching^a^ ^,^ ^b^.

Age^c^	Breast muscle glycogen content, mg/g	Liver glycogen content, mg/g
E23	3.79 ± 0.41^b^	6.06 ± 0.71^d^
E27	5.00 ± 0.77^a^	6.86 ± 0.83^cd^
DOH	1.73 ± 0.17^c^	4.98 ± 0.44^e^
D1	2.07 ± 0.17^c^	7.31 ± 0.93^c^
D4	1.83 ± 0.13^c^	21.57 ± 0.74^b^
D7	1.63 ± 0.09^c^	26.61 ± 0.72^a^

^a-e^Different superscripts within a column indicate a significant difference (*p* < 0.05).

a The data are presented as the means ± standard deviation.

b Value presents the means of six samples (values from the three birds were pooled to form one sample) per sampling timepoint (*n* = 6).

c Age refers to embryonic day 23 (E23), day 27 (E27), day of hatch (DOH; after hatch but before feeding), and day 1 (D1), 4 (D4), and 7 (D7) post hatching.

Glycogen is preferentially used as an energy source by the embryo due to its high-energy supply efficiency (Moran, 2007). However, if glycogen depletion results in insufficient energy supply in embryos, the embryos tend to mobilize muscle protein for gluconeogenesis to compensate for energy shortage (Moran, 2007; Ebrahimnezhad et al., 2011; Gonçalves et al., 2013), which will therefore lead to breast muscle atrophy and growth retardation (Sklan, 2001; EI-Husseiny et al., 2008; Kornasio et al., 2011). The breast muscle is not only an important economic parameter but also plays an important role in metabolism, which is mainly due to its relatively large size and glycogen storage capacity (De Oliveira et al., 2008; Saki et al., 2014). Although the contents of glycogen per unit mass of breast muscle are less than those in the liver, it accounts for the largest amount of total glycogen stored in the body (Uni et al., 2005). As reported, Chen et al. (2009) noted that pectoralis mass decreased with the age of duck embryos during the period of late-term hatching. In the present study, the relative weight of breast muscle decreased with the age of the embryo (Table 3). Additionally, it was observed that the glycogen reserve in breast muscle and the liver was positively correlated with the relative weight of the breast muscle (Table 5). Therefore, the reduction of breast muscle mass indicated that glycogen consumption occurred in the late stage of embryo development, which was the result of physiological activities in the late stage of embryo adaptation.

**TABLE 5:** Partial correlations among body weight, small intestine, and breast muscle parameters, liver and breast muscle contents, and breast muscle-related gene expression.

Variable	Breast muscle parameter	Diameter of myofiber	Breast muscle glycogen content	Liver glycogen content
Breast muscle parameter	1.000	0.116	−0.234	0.664^***^
Diameter of the myofiber		1.000	0.609^***^	0.571^***^
Breast muscle glycogen contents			1.000	0.451^**^
Liver glycogen contents				1.000

*p* < 0.05, *p* < 0.01, *p* < 0.001.

Additionally, in this study, the glycogen content in the breast muscle increased before hatching (Table 4). Similarly, Tazawa et al. (1992) noted that the storage of muscle glycogen in chicks increased from embryonic days 14–19. This may be due to the increase in muscle glycogen storage before hatching, which was beneficial for fueling muscle contractions during pipping and emergence (Pearce, 1977; Tazawa et al., 1992). Moran (2007) also reported that muscles exclusively used glycogen-derived glucose for rapid and intense contraction before hatching, which was required for shell perforation and chick emergence. Therefore, it was essential to increase the glycogen content in breast muscles before hatching to help goslings break the shell. Moreover, the increase in myofiber diameter before hatching and the positive correlation between myofiber diameter and liver glycogen content observed in this study also supported this statement (Tables 3, 5, 6).

**TABLE 6:** Representative myofiber H&E stain slice of goslings between embryonic day 23 to day 7 post hatching^a^ ^,^ ^b^.

Diameter of myofiber, 4 × 10		
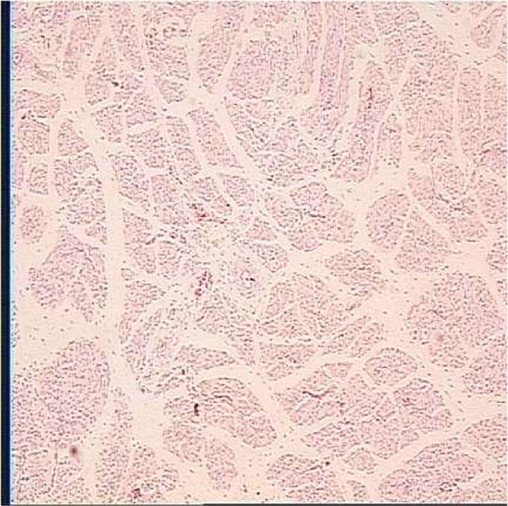	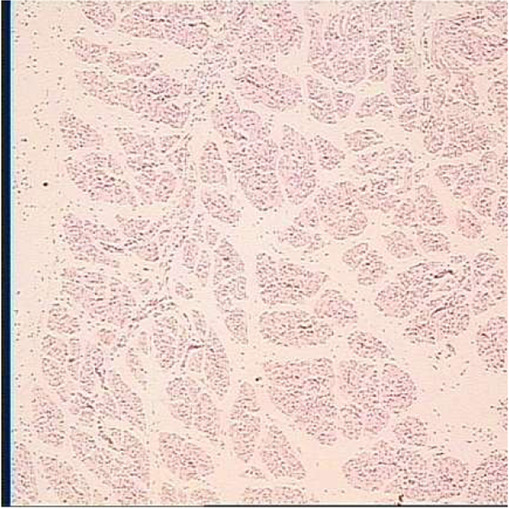	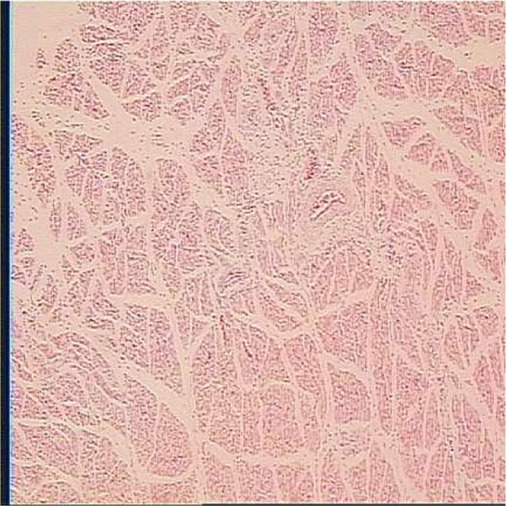
E23	E27	DOH
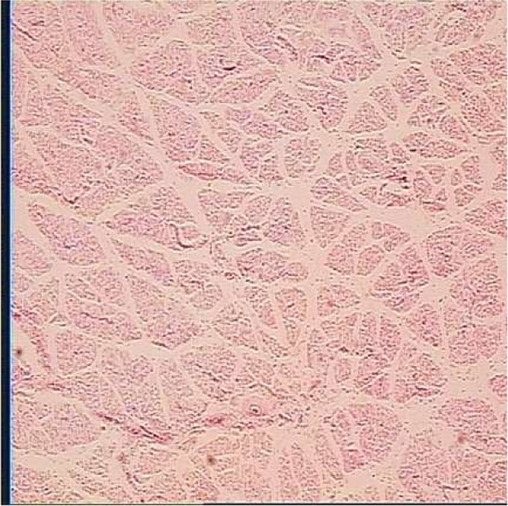	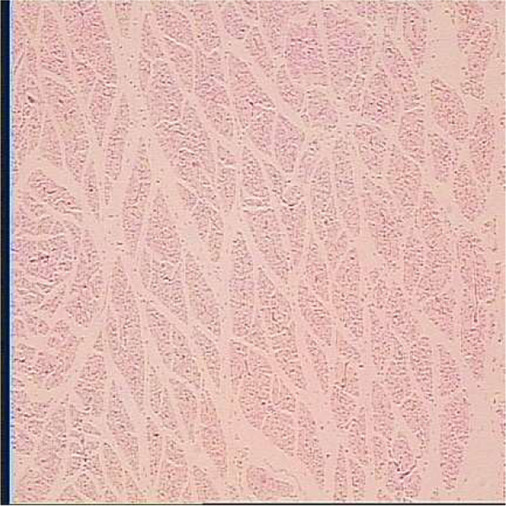	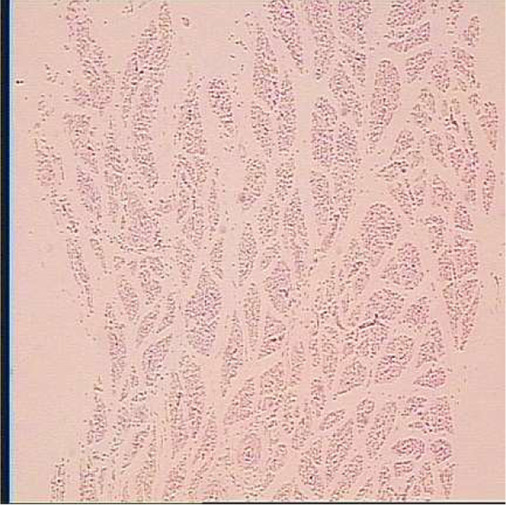
D1	D4	D7

a Age refers to embryonic day 23 (E23), day 27 (E27), day of hatch (DOH; after hatch but before feeding), and day 1 (D1), 4 (D4), and 7 (D7) post hatching.

b One of five images from each sample (in total 18 embryos were used to measure myofiber parameters in each sampling timepoint).

After hatching, the contents of liver glycogen increased with age (Table 4), which was confirmed by the studies of Christensen et al. (20011) and Moran (2007), who noted that the glycogen storage decreased to a very low level at hatching and started to increase when the newly hatched chick was able to fully obtain oxygen and make full use of the fat stored in the yolk sac and glycolytic muscles. Therefore, the increase in liver glycogen content indicated an enhancement in the capacity of nutrient utilization of goslings.

On the other hand, embryonic and postnatal muscle development mainly depends on the total number and cross-sectional area of myofibers (Zhang et al., 2021). However, the total number of myofibers is normally fixed in the embryonic stage and does not increase during the postnatal and early post-hatching period for birds (Li et al., 2020; Xu et al., 2021; Zhang et al., 2021). Therefore, myofiber diameter is the main factor that determines muscle yield in animals (Scheuermann et al., 2003; Zhang et al., 2021). In this study, the diameter of the myofiber decreased during post-hatching development, which was consistent with the decrease in the relative weight of the breast muscle observed in this study (Tables 3, 6). The results observed in this study were also supported by the study of Moore et al. (2005), who noted that the cross-sectional area of turkey embryos decreased as they approached hatching age. In addition, the diameter of the myofiber and the relative weight of breast muscle were positively correlated with the content of liver glycogen (Table 5). Therefore, we speculated that the reduction in myofiber diameter and the relative weight of the breast muscle were probably related to consumption of glycogen reserves during development.

**FIGURE 1:**
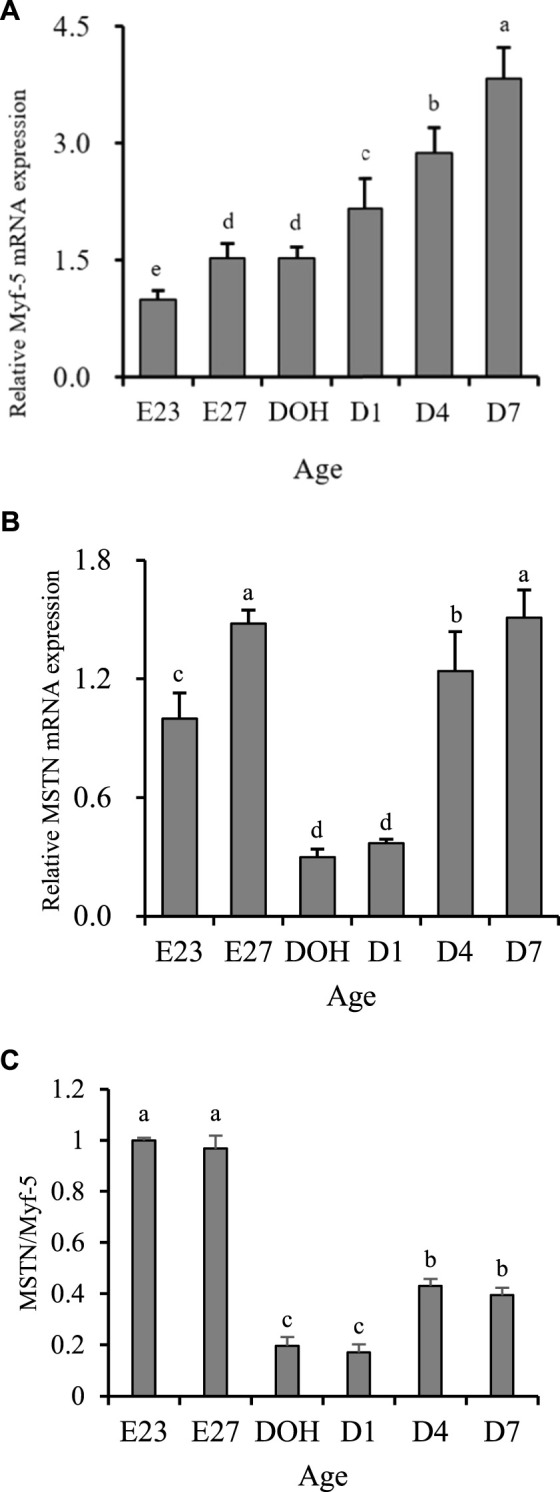
Breast growth-related gene expression of geese on embryonic day 23 (E23), day 27 (E27), day of hatch (DOH; after hatch but before feeding), and day 1 (D1), 4 (D4), and 7 (D7) post hatching. ^a-e^Different superscripts between columns indicate significant difference (*P* < 0.05). The data are presented as the mean ± standard deviations. The value presents the means of six samples (values from the three birds were pooled to form one sample) per sampling timepoint (*n* = 6). Sampling timepoint of E23 was used as the untreated control timepoint (value = 1). ^a-e^Different superscripts within a column indicate a significant difference (*p* < 0.05).

Myofiber enlargement or hypertrophy events are related to fusion of satellite cells and myofiber (Allen and Boxhorn, 1989). Many regulatory factors can regulate this process. Among them, Myf-5 and MSTN are important factors in regulating the development of muscles (Wu et al., 2016). Myf-5 could promote the mitotic activity of satellite cells (Dias et al., 1994). MSTN could inhibit cell proliferation and cell growth by controlling the transition from G (1)-phase to S-phase in the cell cycle and therefore reduce the hyperplasia of myoblasts (Thomas et al., 2000). In terms of biological effects caused by the expression of Myf-5 and MSTN, high expression of Mfy-5 was beneficial to muscle development, while high expression of MSTN was disadvantageous. In this study, the increase in myofiber diameter was observed from embryonic days 23–27 and days 1–7 post hatching, while the decrease was observed from embryonic day 27 to day 1 post hatching. The development pattern of myofiber diameter was partially consistent with the expression of Myf-5 but completely opposite to the expression of MSTN (Figures 1A,B). However, its development was matched with the changes in the MSTN/Myf-5 ratio (Figure 1C). Similar contradictory results have also been reported in mice exhibiting muscle-specific overexpression of MSTN. Reisz-Porszasz et al. (2003) observed that the increase in MSTN protein expression in the skeletal muscle has no significant effect on the skeletal muscle mass of female transgenic mice. Additionally, some contradictory elements have been published concerning the effect of MSTN inhibition on myogenic factor levels in zebrafish (Xu et al., 2003; Amali et al., 2004). Similarly, results of breast muscle parameters observed in this study indicated that the highest relative weight of breast muscle was present before hatching, while it gradually decreased during the post-hatching period (Table 3). However, gene expression was not the same. We observed that the expression of Myf-5 remained at a low level before hatching but gradually increased post-hatching (Figure 1A). In addition, the expression of MSTN during embryonic days 23–27 remained high and remained low during hatching day to day 1 post hatching (Figure 1B). But, we observed that the development of breast muscle parameters matched the changes of the MSTN/Myf-5 ratio (Figure 1C). Similar to the development of the breast muscle, the ratio of MSTN/Myf-5 remained at a high level before hatching but at a relatively low level post hatching (Figure 1C). Therefore, we thought that the ratio of MSTN/Myf-5 could potentially be more important in regulating muscle growth than the relative expression of Myf-5 and MSTN analyzed independently.

## Conclusion

This is the first study to investigate the development patterns of breast muscle and glycogen reserves in goslings during pre- and post-hatching periods. We discovered that the body weight and the small intestine developed rapidly throughout pre- and post-hatching periods, which led to considerable energy catabolism, resulting in the reduction of glycogen reserves and therefore impairing the breast muscle parameters. In addition, the expression of Myf-5 and MSTN plays a key role in the regulation of breast muscle development. The ratio MSTN/Myf-5 is capable of being used as an indicator of myofiber hypertrophy. After hatching, liver glycogen reserves increased rapidly to support its further development. It is suggested that some specific strategies should be implemented to improve the energy levels of embryos, which may aid in the development of goslings.

## Data Availability

The raw data supporting the conclusion of this article will be made available by the authors, without undue reservation.
